# DNA Polymerase Eta Stabilizes Mutant p53 Protein to Increase Tumor Cell Growth

**DOI:** 10.3390/cells15201881

**Published:** 2026-10-15

**Authors:** Jin Zhang, Yang Shi, Xinbin Chen

**Affiliations:** Comparative Oncology Laboratory, Weill School of Veterinary Medicine and School of Medicine, University of California, Davis, CA 95616, USA; ysyshi@ucdavis.edu

**Keywords:** PolH, mutant p53, SKP2, chemoresistance, protein stability

## Abstract

DNA polymerase eta (PolH) is a specialized translesion synthesis DNA polymerase that enables replication across damaged DNA and contributes to cellular tolerance of genotoxic stress. Although PolH has been implicated in chemoresistance, its role in oncogenic signaling remains poorly understood. Here, we identify an unexpected role of PolH in tumor cell growth via mutant p53. We found that overexpression of PolH increases the level of mutant p53 protein. By contrast, knockdown or knockout of PolH inhibits mutant p53 expression by decreasing the half-life of mutant p53 protein. We also found that knockdown or knockout of PolH leads to increased expression of SKP2. As a key E3 ubiquitin ligase component of the SCF complex, we showed that overexpression of SKP2 decreases whereas knockdown of SKP2 increases mutant p53 expression. Moreover, we showed that knockdown of SKP2 abrogates the ability of PolH to regulate mutant p53 expression. To determine the role of SKP2 in mediating mutant p53 proteasome degradation, we showed that SKP2 interacts with mutant p53(R237H). We also found that wild-type but not F-box-deleted SKP2 poly-ubiquitinates mutant p53(R273H). Importantly, we showed that tumor sphere formation is inhibited by knockdown or knockout of PolH, which is further inhibited by knockdown of mutant p53, supporting a cooperative role for PolH and mutant p53 in tumor cell growth. Collectively, these observations indicate that PolH is a previously unrecognized regulator of mutant p53 via SKP2 and that the PolH–mutant p53 pathway can be explored as a therapeutic strategy for mutant p53-driven cancers.

## 1. Introduction

Chemotherapy has long been a first-line treatment for many types of cancers. Chemo-drugs, such as cisplatin, are designed to kill tumor cells by inducing DNA damage response. In most cases, patients initially show a positive response to treatment, but later they often develop drug resistance, leading to disease progression. Thus, chemoresistance is a major cause of treatment failure [1,2]. Depending on the time, chemoresistance can be either intrinsic, existing before treatment, or acquired, developing during therapy. Several mechanisms have been proposed to contribute to chemoresistance, one of which is the ability of tumor cells to tolerate chemotherapy-induced DNA damage through translesion DNA synthesis (TLS) [3,4,5,6,7]. TLS is a process mediated by low-fidelity DNA polymerases that enables DNA replication across lesions [8,9]. Unlike high-fidelity DNA polymerases, TLS polymerases possess flexible active sites that can accommodate distorted DNA structures, allowing them to bypass specific DNA lesions and resume DNA synthesis [10]. As an error-prone DNA replication process, TLS can introduce mutations in normal and tumor cells as well as in relapsed malignancies [11,12]. Recently, in addition to their canonical role in lesion bypass, TLS polymerases were also found to play critical roles in DNA replication, DNA repair, and gene transcription [9,13,14].

Most TLS DNA polymerases belong to the Y-family of DNA polymerases, including PolH, PolK, PolI, and Rev1 [15], as well as the B-family DNA polymerase Pol Z (consisting of two subunits, Rev3 and Rev7) [16]. Among them, PolH is unique in its ability to efficiently and accurately bypass UV-induced cyclobutane pyrimidine dimers (CPDs) [17]. The biological significance of PolH is underscored by the hereditary disease xeroderma pigmentosum variant (XPV), which is caused by germline mutations that inactivate PolH [18,19]. However, despite its ability to accurately bypass CPDs, PolH can replicate across DNA lesions such as cisplatin-induced 1,2-d (GpG) adduct (Pt-GG) in an error-prone manner [20,21,22]. Indeed, high levels of PolH were found after platinum-based chemotherapy and associated with poor survival of cancer patients including those with non-small-cell lung cancer (NSCLC) [7,23,24]. Consistent with this, we previously showed that loss of PolH sensitizes lung cancer cells to cisplatin resistance treatment [25]. However, whether PolH contributes to the regulation of oncogenic proteins remains poorly understood.

The tumor suppressor p53 is often referred to as the “guardian of the genome” [26,27]. The importance of p53 in tumor suppression is underscored by its ability as a transcription factor to regulate a series of downstream target genes [28]. As one of the most commonly mutated genes in human cancers, loss of p53 function is known to play a central role in tumor development [29]. Interestingly, unlike most tumor-suppressor genes, which are mainly inactivated as a result of deletion or truncation, the vast majority of tumor-derived p53 mutations are missense mutations and clustered within the central DNA-binding domain [30]. Notably, some mutants acquire new oncogenic properties, generally referred to as gain-of-function, such as the ability to promote cell proliferation, metastasis, and chemoresistance [31,32]. Thus, mutant p53 is considered a therapeutic vulnerability for cancer treatment.

While investigating the role of PolH in chemoresistance, we made the unexpected discovery that PolH regulates mutant p53 protein stability and, subsequently, tumor cell growth. We found that loss of PolH markedly reduces mutant p53 protein levels, which is attributable to a shortened mutant p53 protein half-life. Mechanistically, loss of PolH increases expression of SKP2, a key component of the SCF E3 ubiquitin ligase, which subsequently promotes proteasomal degradation of mutant p53. Furthermore, we showed that tumor sphere formation is inhibited by loss of PolH, which can be further inhibited by additional knockdown of mutant p53. Together, these data revealed a previously unrecognized role of PolH in regulating mutant p53 protein stability via SKP2, suggesting that targeting PolH can be explored as a therapeutic strategy for mutant p53-driven cancers.

## 2. Materials and Methods

### 2.1. Reagents

Anti-PolH (Cat# A301-231A) was purchased from Foris Life Sciences (Boston, MA, USA). Anti-p53 (Cat# sc-126), anti-Vinculin (Cat# sc-73614), and anti-actin (Cat# sc-58673) were purchased from Santa Cruz Technology (Dallas, TX, USA). Anti-GAPDH (Cat# 5174S) and Anti-SKP2 (Cat # 4358S) were purchased from Cell Signaling Technology (Danvers, MA, USA). Mouse anti-Flag (Cat# F1804) and cycloheximide (Cat# C1988) were purchased from Sigma (St. Louis, MO, USA). Rabbit anti-Flag (Cat# 20543-1-AP) was purchased from Proteintech (Rosemont, IL, USA). Protein A/G magnetic beads (Cat# HY-k0202), proteinase inhibitor cocktail (Cat#HY-k0011), and MG132 (Cat# HY-13259) were purchased from MedChemExpress (Monmouth Junction, NJ, USA). The WesternBright Sirius HRP substrate (Cat# K12043-D20) was purchased from Advansta (Foster City, CA, USA). JetPRIME transfection reagent (Cat# 101000046) was purchased from Polyplus (Paris, France).

### 2.2. Cell Culture

SW480, MIA-PaCa2, H1975, A549, and 293T cell lines were acquired from ATCC (Manassas, VA, USA) and utilized at passages below 20. SW480, MIA-PaCa2, and the cells and their derivatives were cultured in DMEM (Dulbecco’s modified Eagle’s medium, Invitrogen, Waltham, MA, USA) supplemented with 10% fetal bovine serum. H1975 and CL1 were cultured in RPMI-1640 medium supplemented with 10% fetal bovine serum (Hyclone, Logan, UT, USA). Additionally, p53 status in these cell lines is shown in Table 1.

### 2.3. Plasmids

pCDNA3-HA-PolH and pCDNA3-HA-Ub vectors were generated previously [33]. pCDNA3-Flag-SKP2 and pCDNA3-Flag-SKP2(ΔFbox) vectors were purchased from Genscript. pCDNA3-p53(R273H) was generated previously [34].

### 2.4. SiRNA Transfection

SiRNA transfection was performed using RNAiMAX (Invitrogen, Waltham, MA, USA) according to the user manual. The sequence for scrambled siRNA was 5′-GCA GUG UCU CCA CGU ACU AdTdT-3′. The sequences for p53 siRNA were 5′-GCA CAG AGG AAG AGA AUC UdTdT-3′ and 5′-GAA AUU UGC GUG UGG AGU AdTdT-3′.

### 2.5. Western Blot Analysis

Western blotting was performed as previously described [35]. Briefly, whole-cell lysates were separated by 8–13% SDS gels, which were then transferred onto nitrocellulose membranes. Membranes were incubated with a primary antibody at 4 °C overnight, followed by extensive washing with PBST and then incubating with the appropriate secondary antibody at 4 °C for 4 h. To visualize the protein, the membrane was incubated with enhanced chemiluminescence and the signals were detected with VisionWorks LS software (version 8.0; Analytik Jena, Jena, Germany).

### 2.6. Immunoprecipitation

For immunoprecipitation analysis, cells were lysed in IP lysis buffer (0.5% NP-40, 50 mM Tris-HCl (pH 8.0), 150 mM NaCl, and 1 mM EDTA) supplemented with a protease inhibitor cocktail. Cell lysates were sonicated and centrifuged at 13,000 rpm for 15 min at 4 °C. The resulting supernatants were incubated with 1 μg of primary antibody or IgG and protein A/G magnetic beads at 4 °C overnight. The next day, the beads were washed 8 times with IP lysis buffer, followed by Western blot analysis to detect immunocomplexes.

### 2.7. In Vivo Ubiquitin Assay

In vivo ubiquitination assay was performed as described [36]. Briefly, 293T cells were transfected with plasmids expressing p53 and HA-tagged ubiquitin with or without a plasmid expressing Flag-tagged SKP2 for 24 h. The next day, the cells were treated with 5 μM MG132 for 6 h and cell lysates were collected. Cell lysates were then immunoprecipitated with anti-HA antibody followed by Western blot with p53 antibody to detect poly-ubiquitinated p53.

### 2.8. Total RNA Isolation, RT-PCR and Quantitative PCR Analyses

Total RNA was isolated with Trizol reagent as described according to the user manual (ThermoFisher Scientific, Waltham, MA, USA). For RT-PCR analysis, 3 μg of total RNA was used to synthesize cDNA using RevertAid reverse transcriptase according to the manufacturer’s manual. Next, the cDNA was then used for PCR analysis using the following program: (i) 94 °C for 5 min, (ii) 94 °C for 45 s, (iii) 58 °C for 45 s, (iv) 72 °C for 45 s, and (v) 72 °C for 10 min. From steps 2 to 4, the cycle was repeated 28 times for SKP2 and PolH, 25 times for p53, and 22 times for actin, HPRT1 and GAPDH. For Quantitative PCR (qPCR) analysis, 20 μL reactions were prepared using 2X SYBR Green Master Mix, 5 μM primers, and template cDNA. Reactions were run on a StepOnePlus(Version 2.3) Real-Time PCR System (Applied Biosystems, Waltham, MA, USA) using a two-step cycling program: 95 °C for 15 min, followed by 40 cycles of 95 °C for 15 s, 60 °C for 30 s, and 68 °C for 30 s. A melt curve (57–95 °C) was generated at the end of each run to verify specificity. The primers for SKP2 were a forward primer, 5′-TTG CCC TGC AGA CTT TGC TA-3′, and a reverse primer, 5′-ACC ATC TGG CAC GAT TCC AA-3′. The primers for PolH were a forward primer, 5′-TGA GAT GGA GTC TCG CTG TG-3′, and a reverse primer, 5′-AAA ATT AGC TGG GCA TGG TG-3′. The primers for p53 were a forward primer, 5′-CCC AGC CAA AGA AGA AAC CA-3′, and a reverse primer, 5′-GTT CCA AGG CCT CAT TCA GCT-3′. The primers for actin were a forward primer, 5′-CTG AAG TAC CCC ATC GAG CAC GGC A-3′, and a reverse primer, 5′-GGA TAG CAC AGC CTG GAT AGC AAC G-3′. The primers for GAPDH were a forward primer, 5′-AGC CTC AAG ATC ATC AGC AAT G-3′ and a reverse primer, 5′-ATG GAC TGT GGT CAT GAG TCC TT-3′. The primers for HPRT1 were a forward primer, 5′-TAT GGC GAC CCG CAG CCC T-3′, and a reverse primer, 5′-CAT CTC GAG CAA GAC GTT CAG-3′.

### 2.9. Tumor Sphere Assay

Cells were cultured in MammoCult™ (Stemcell, Cambridge, MA, USA) media per the manufacturer’s guidelines. Approximately 20,000 cells were seeded per well in 6-well ultra-low adherent plates for 2 weeks. For each experimental group, approximately 150 tumor spheres were quantified across multiple randomly selected fields using an inverted light microscope. All assays were performed in three independent biological replicates to ensure statistical reliability.

## 3. Results

### 3.1. PolH Is Required for Mutant p53 Expression

**Figure 1:**
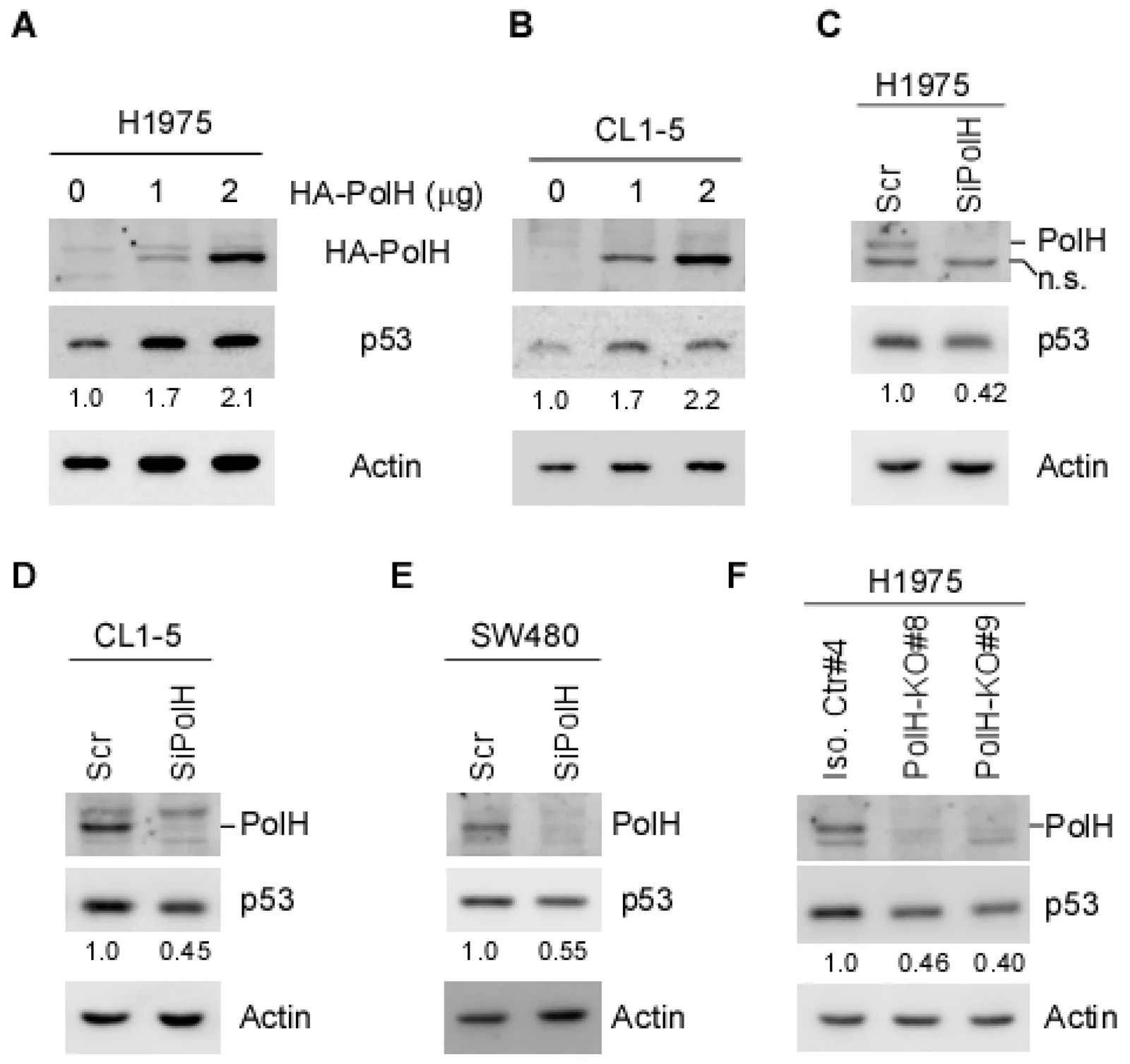
PolH is required for mutant p53 expression (**A**,**B**). H1975 (**A**) and CL1-5 (**B**) cells were transfected with 0–2 μg of PolH-expressing plasmids, followed by western blot analysisto detect the expression of PolH, mutant p53 and actin. p53 protein levels were normalized to control cells (arbitrarily set to 1.0), and relative fold changes are shown below each lane. (**C**–**E**) H1975 (**C**), CL1-5 (**D**) and SW480 (**E**) cells were transiently transfected with scrambled siRNA or PolH siRNA for 3 days, followed by western blot analysis to measure the levels of PolH, p53 and actin. p53 protein levels were normalized to control cells (arbitrarily set to 1.0), and relative fold changes are shown below each lane. (**F**) The levels of PolH, p53 and actin proteins were measured in isogenic control and PolH-KO H1975 cells. p53 protein levels were normalized to control cells (arbitrarily set to 1.0), and relative fold changes are shown below each lane.

Previous studies have shown that both PolH and mutant p53 contribute to chemoresistance in several types of cancers [24,25,37,38]. However, it is uncertain whether there is any crosstalk between PolH and mutant p53. In this regard, we sought to determine whether PolH regulates mutant p53 expression in H1975 and CL1-5 non-small-cell lung cancer cells. H1975 harbors mutant p53R273H [39], whereas CL1-5 carries mutant p53R248W [40]. Briefly, H1975 and CL1 cells were transiently transfected with a DNA plasmid expressing HA-tagged PolH, followed by western blot to detect mutant p53 expression. We found that ectopic expression of PolH led to increased expression of mutant p53 in both H1975 and CL1-5 cells (Figure 1A,B). To verify this, PolH was depleted in H1975 cells using siRNA. We found that knockdown of PolH resulted in decreased expression of mutant p53 in H1975 cells (Figure 1C). Consistent with this, we showed that mutant p53 was decreased by knockdown of PolH in both CL1-5 and SW480 cells (Figure 1D,E). SW480 cells carry a mutant p53 (R273H/P309S) [41]. To further verify this, isogenic control and PolH-KO H1975 cells were used to measure mutant p53 expression. We found that knockout of PolH led to decreased mutant p53 expression (Figure 1F). Together, these data suggest that PolH is required for mutant p53 expression.

### 3.2. PolH Is Required for Maintaining Mutant p53 Protein Stability

**Figure 2:**
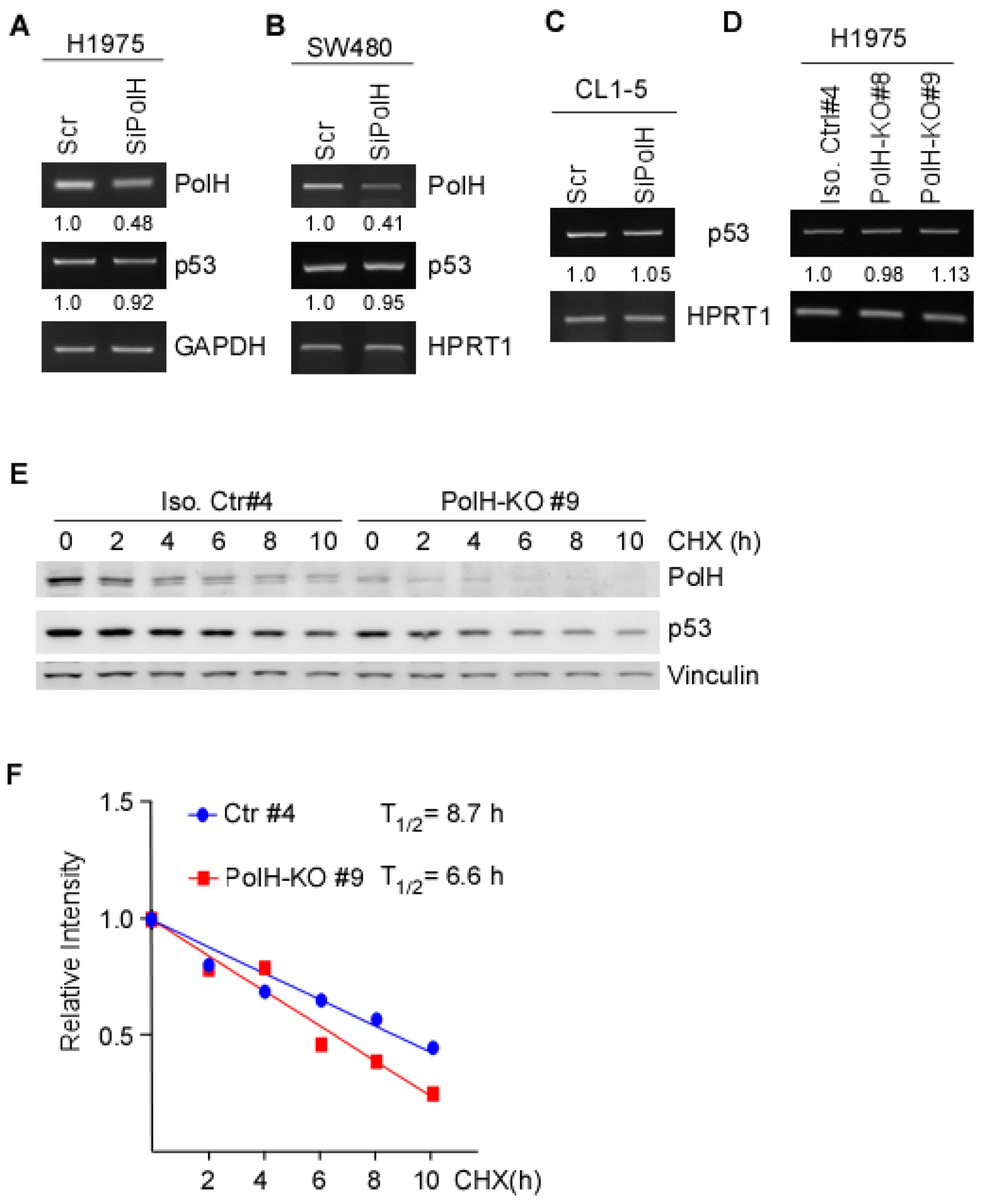
PolH is required for maintaining mutant p53 protein stability. (**A**) H1975 cells were transiently transfected with scrambled and PolH siRNA for 3 days, followed by RT-PCR to detect the levels of PolH, p53 and GAPDH transcripts. Relative levels of PolH and p53 transcripts were normalized to GAPDH or HPRT1 and the relative fold change is indicated below each lane. (**B**,**C**) The levels of PolH, p53, and HPRT1 transcripts were measured in SW480 (**B**) and CL1-5 (**C**) cells transfected with scrambled or PolH siRNA for 3 days. The relative levels of p53 transcripts were normalized to HPRT1 and the relative fold change was indicated below each lane. (**D**) The levels of p53 and HPRT1 transcripts were measured in isogenic control and PolH-KO H1975 cells. (**E**) Isogenic control and PolH-KO H1975 cells were treated with cycloheximide from 0 to 10 h, followed by western blot to detect the expression of PolH, p53 and Vinculin. (**F**) Quantification of mutant p53 protein half-life shown in (**E**). The average densitometric values from three independent experiments were plotted as a percentage of p53 remaining as a function of time after CHX treatment. The p53 half-life was calculated using linear regression analysis.

To explore the underlying mechanism by which PolH modulates mutant p53 expression, the levels of PolH, mutant p53 and GAPDH transcripts were measured in H1975 cells with or without PolH knockdown. We found that the level of PolH transcript was decreased upon transfection of PolH siRNA (Figure 2A, PolH panel). However, the level of mutant p53 transcript was not altered by knockdown of PolH (Figure 1A, p53 panel). To verify this, SW480 and CL1-5 cells were used to knock down PolH. We found that knockdown of PolH did not alter the level of mutant p53 transcripts (Figure 2B,C). Consistent with this, we found that the level of p53 transcript was not changed in PolH-KO H1975 cells as compared to that in isogenic control cells (Figure 2D). These data let us postulate that PolH modulates mutant p53 protein stability. To test this, isogenic control and PolH-KO H1975 cells were treated with cycloheximide from 0 to 10 h, followed by measurement of mutant p53 protein. We found that loss of PolH led to a reduction in mutant p53 protein half-life from 8.7 h to 6.6 h (Figure 2E,F).

### 3.3. SKP2 Is Regulated by PolH

**Figure 3:**
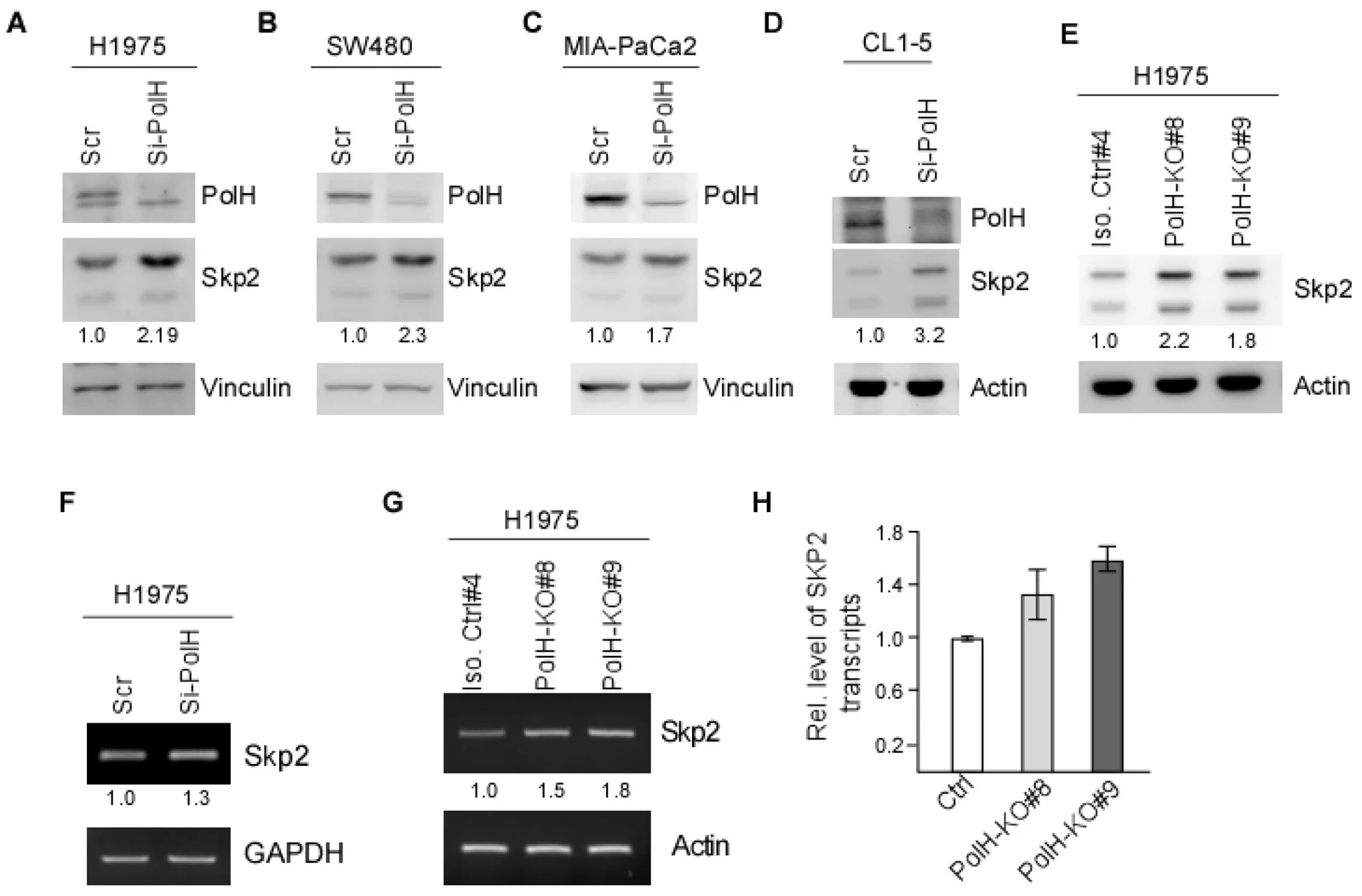
SKP2 is regulated by PolH (**A**–**D**). H1975 (**A**), SW480 (**B**), MIA-PaCa2 (**C**), and CL1-5 (**D**) cells were transfected with scrambled and PolH siRNA for 3 days, followed by western blot analysis to detect PolH, Skp2 and actin, Vinculin proteins. Protein levels were normalized to the control, and the relative fold change is indicated below each lane. (**E**) The levels of Skp2 and actin proteins were measured in isogenic control and PolH-KO H1975 cells. Protein levels were normalized to the control, and the relative fold change is indicated below each lane. (**F**) H1975 cells were transfected with scrambled and PolH siRNA for 3 days, followed by RT-PCR analysis to measure Skp2 and actin transcripts. The levels of SKP2 transcripts were normalized to GAPDH, and the relative fold change is indicated below each lane. (**G**) The levels of Skp2 and actin transcripts were measured in isogenic control and PolH-KO H1975 cells. The levels of SKP2 transcripts were normalized to actin, and the relative fold change is indicated below each lane. (**H**) qPCR analysis was performed using isogenic control and PolH-KO H1975 cells.

To investigate how PolH regulates the half-life of mutant p53 protein, we sought to identify an E3 ligase that may be involved in this process. To this end, we tested a few E3 ligases, including S-phase kinase-associated protein 2 (SKP2). SKP2 is an F-box protein of the SCF ubiquitin ligase complex, which plays an important role in the regulation of cell-cycle progression [42]. First, we examined SKP2 expression in H1975 cells transfected with scrambled or PolH siRNA. Interestingly, we found that the level of SKP2 protein was increased upon knockdown of PolH (Figure 3A). Likewise, we found that knockdown of PolH led to increased expression of SKP2 in SW480, Mia-PaCa2, and CL1-5 cells (Figure 3B–D). Moreover, we found that the level of SKP2 protein was increased in PolH-KO H1975 cells as compared to isogenic control H1975 cells (Figure 3E). In addition, we examined whether SKP2 expression was altered by knockout of PolH in A549 cells, which contain wt p53. We found that SKP2 expression was moderately increased by PolH-KO (Supplemental Figure S1, SKP2 panel). Notably, the level of wild-type p53 was slightly increased by PolH-KO in A549 cells (Supplemental Figure S1, p53 panel). Further, we analyzed the level of SKP2 transcripts in H1975 cells with or without PolH knockdown or knockout. We found that SKP2 is moderately increased upon PolH knockdown or knockout (Figure 3F,G). To further verify that SKP2 transcripts were increased by knockout of PolH, qPCR analysis was performed with isogenic control and PolH-KO H1975 cells. We found that the level of PolH transcripts was increased in PolH-KO H1975 cells as compared to isogenic control cells (Figure 3H). Together, these data indicated that SKP2 expression is likely repressed by PolH at least in part through transcriptional regulation.

### 3.4. SKP2 Inhibits Mutant p53 Expression

**Figure 4:**
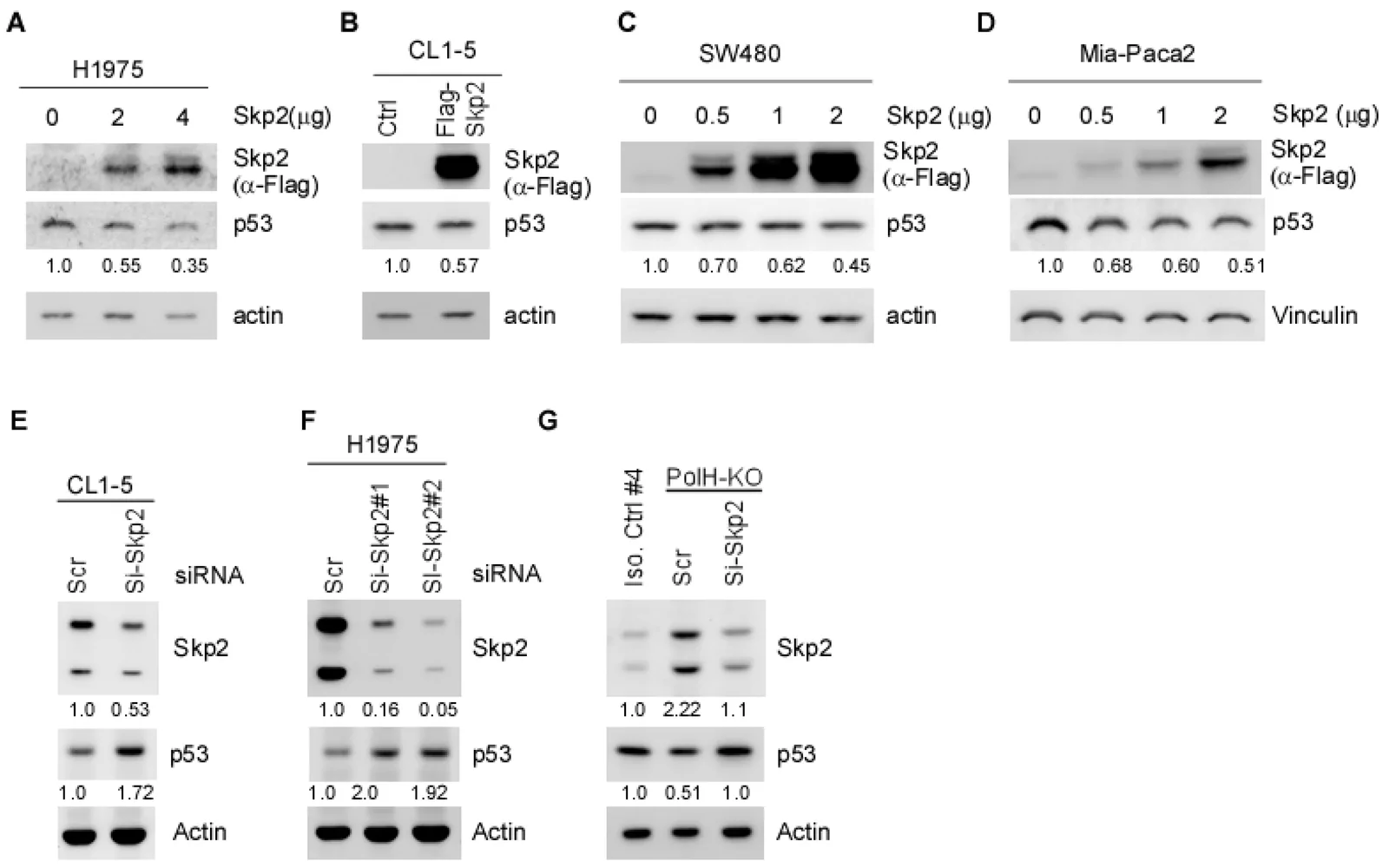
SKP2 inhibits mutant p53 expression (**A**–**D**). H1975 (**A**), CL1-5 (**B**), SW480 (**C**), and MIA-PaCa2 (**D**) cells were transfected with an empty vector or a vector expressing Flag-tagged SKP2 for 24 h, followed by western blot analysis to measure the levels of Skp2, p53 and actin. Protein levels were normalized to actin or Vinculin, and the relative fold change is indicated below each lane. (**E**,**F**) CL1-5 (**E**) and H1975 (**F**) cells were transfected with scrambled or SKP2 siRNA for 3 days, followed by western blot to detect the levels of Skp2, p53, and actin proteins. Protein levels were normalized to actin, and the relative fold change is indicated below each lane. (**G**) The levels of SKp2, p53, and actin proteins were measured in isogenic control H1975 cells or PolH-KO H1975 cells with or without Skp2 knockdown. Protein levels were normalized to actin, and the relative fold change is indicated below each lane.

Previously, SKP2 was found to block the interaction between p300 and wild-type p53, thereby inhibiting apoptosis [43]. But it is not certain whether it can regulate mutant p53 expression. To test this possibility, SKP2 was transiently transfected into H1975 cells, followed by detection of mutant p53 protein. We found that ectopic expression of SKP2 inhibited mutant p53 expression in H1975 cells (Figure 4A). Similarly, overexpression of SKP2 suppressed mutant p53 expression in CL1-5, SW480, and MIA-PaCa2 cells (Figure 4B–D). To verify this observation, we found that mutant p53 expression was increased by knockdown of SKP2 in both CL1-5 and H1975 cells (Figure 4E,F). Next, we set out to determine whether SKP2 contributes to the increased mutant p53 expression in PolH-KO H1975 cells with or without knockdown of SKP2. As expected, the level of SKP2 protein was increased in PolH-KO H1975 cells transfected with scrambled siRNA, which was also accompanied by decreased mutant p53 expression (Figure 4G). However, upon knockdown of SKP2 in PolH-KO H1975 cells, mutant p53 expression did not decrease but instead increased (Figure 4G), suggesting that SKP2 plays a key role in PolH-mediated regulation of mutant p53 expression.

### 3.5. SKP2 Promotes Mutant p53 for Proteasomal Degradation

**Figure 5:**
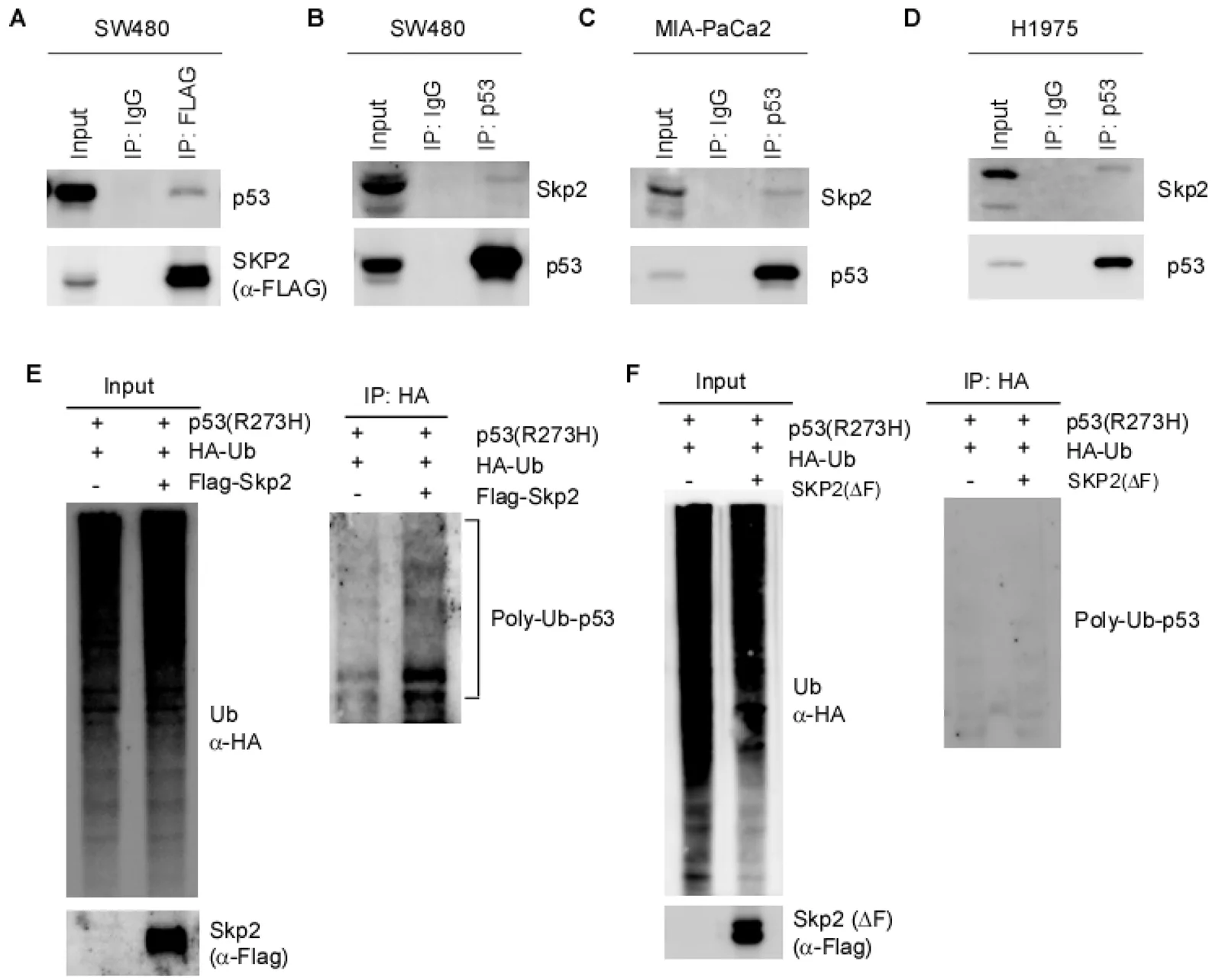
SKP2 promotes mutant p53 for proteasomal degradation. (**A**) SW480 cells were transfected with a plasmid expressing Flag-tagged SKP2 for 24 h. Cell lysates were collected and immunoprecipitated with IgG or Flag antibody. The immunocomplex was subjected to western blot analysis using antibodies against p53 or Flag. (**B**–**D**) SW480 (**B**), MIA-PaCa2 (**C**), and H1975 (**D**) cell lysates were immunoprecipitated with IgG or p53 antibody and the immunocomplex was detected by western blot using antibodies against p53 or Skp2. (**E**,**F**) In vivo ubiquitin assay was performed with 293T cells transfected with plasmids expressing p53(R273H) and HA-tagged ubiquitin with or without a plasmid expressing Flag-tagged Skp2 (**E**) or SKP2(ΔF) (**F**) for 24 h. Left panel: 5% of whole lysates were used as input and subjected to western using antibodies against HA and Flag to detect ubiquitin and Skp2 (**E**) or Skp2(ΔF) (**F**). Right panel: the rest of the cell lysates were then immunoprecipitated with HA antibody and the immunocomplexes were detected by western blot using p53 antibody.

To explore how SKP2 represses mutant p53 expression, we sought to determine whether SKP2 directly interacts with mutant p53 protein. To this end, IP-western blot analysis was performed with SW480 cell lysates expressing Flag-tagged SKP2. We found that when the Flag antibody was used to bring down SKP2, p53 was present in the SKP2 immunocomplexes (Figure 5A), suggesting that mutant p53 is associated with SKP2. To verify this, SW480 cells were used for immunoprecipitation using an antibody against endogenous mutant p53, followed by detection of SKP2. We found that SKP2 was also present in the p53 immunocomplexes (Figure 5B). Moreover, we found that endogenous SKP2 was associated with endogenous mutant p53 in both MIA-PaCa2 and H1975 cells (Figure 5C,D).

Next, we examined whether SKP2 can poly-ubiquitinate mutant p53 by performing an in vivo ubiquitin assay. Briefly, 293T cells were transfected with plasmids expressing p53 and HA-tagged ubiquitin with or without a plasmid expressing Flag-tagged wild-type or F-box-deleted SKP2. The F-box in SKP2 is known to be required for its E3 ligase activity [44]. The cell lysates were then immunoprecipitated with an anti-HA antibody, followed by Western blot with a p53 antibody. We showed that the level of poly-ubiquitinated p53 was markedly increased when co-expressed with Flag-SKP2 (Figure 5E) but not by Flag-SKP2(ΔF) (Figure 5F). Together, these data indicate that SKP2 can poly-ubiquitinate mutant p53.

### 3.6. Mutant p53 Contributes to PolH-Mediated Tumor Cell Growth and Survival

**Figure 6:**
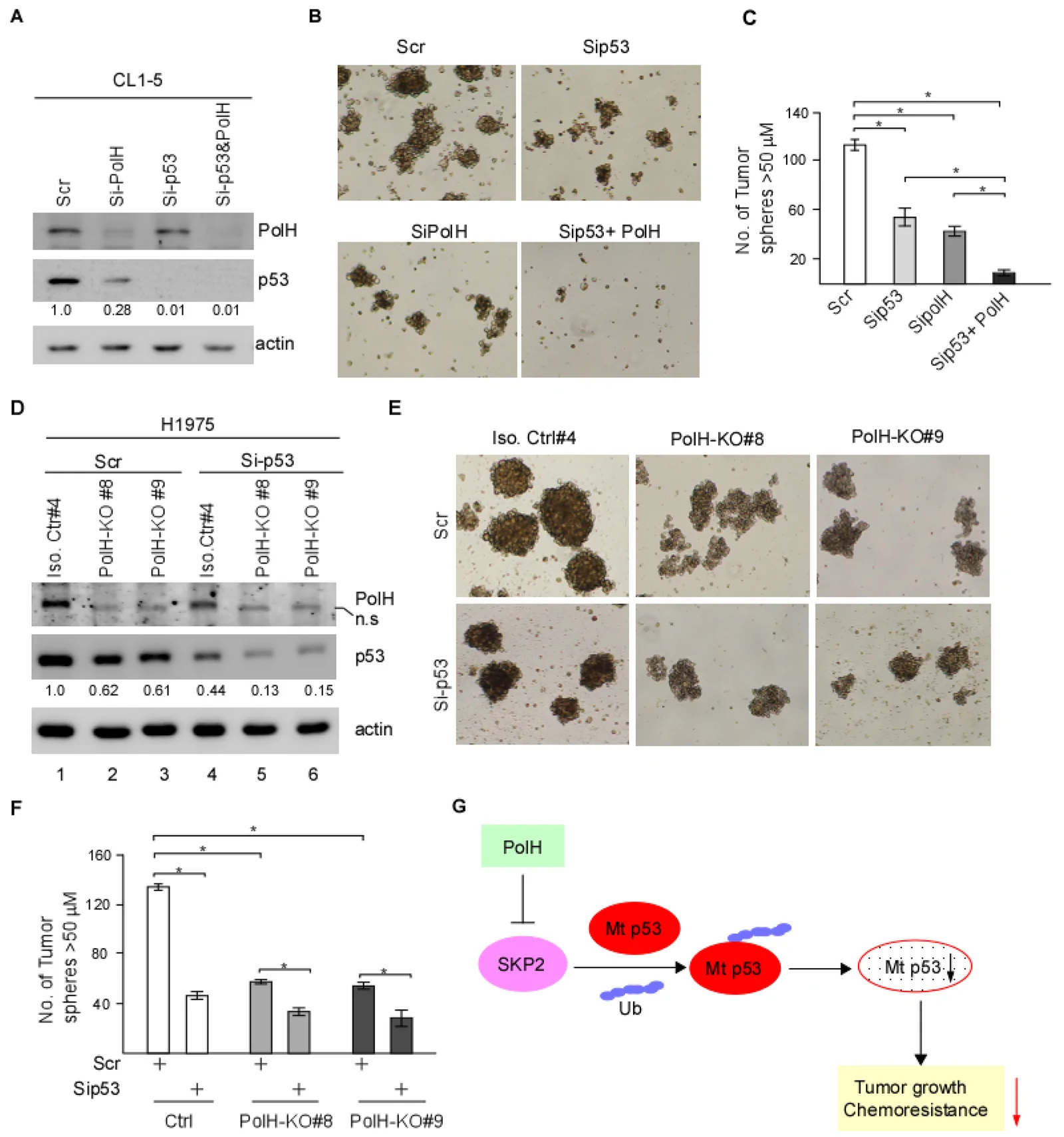
Mutant p53 contributes to PolH-mediated tumor cell growth and survival. (**A**) CL1-5 cells were transiently transfected with scrambled siRNA, PolH siRNA, p53 siRNA, or a combination of PolH and p53 siRNAs for 3 days, followed by western blot analysis to detect PolH, p53 and actin. PolH and p53 protein levels were normalized to actin, and the relative fold change is indicated below each lane. (**B**) Tumor sphere assay was performed with cells treated in (**A**). (**C**) Quantification of tumor spheres larger than 50 μm from (**B**). * indicates that *p* < 0.05 by Student’s *t*-test. (**D**) Isogenic control and PolH-KO H1975 cells were transfected with a scrambled or p53 siRNA for 3 days, followed by western blot to detect PolH, p53 and actin proteins. PolH and p53 protein levels were normalized to actin, and the relative fold change is indicated below each lane. (**E**) Tumor sphere assays were performed with cells treated in (**D**). (**F**) Quantification of tumor spheres larger than 50 μm in (**E**). * indicates that *p* < 0.05 by Student’s *t*-test. (**G**) A model is proposed to elucidate the role of the PolH–mutant p53 pathway in tumor growth and chemoresistance.

Previously, we showed that PolH is required for tumor cell survival [25]. To determine whether mutant p53 plays a role in PolH-mediated tumor cell growth, CL1-5 cells were transiently transfected with a scrambled siRNA or siRNA against PolH and/or mutant p53, followed by tumor sphere assays. As expected, the levels of PolH and mutant p53 proteins were reduced upon transfection of their respective siRNAs (Figure 6A). We found that the number of tumor spheres was decreased by knockdown of PolH or mutant p53, which was further decreased by combined knockdown of both PolH and mutant p53 (Figure 6B,C). To verify this, isogenic control and PolH-KO H1975 cells were transiently transfected with p53 siRNA, followed by a tumor sphere assay. As expected, the level of mutant p53 protein was low in PolH-KO cells, which was further decreased by p53 siRNA in both isogenic control and PolH-KO H1975 cells (Figure 6D). As expected, knockdown of mutant p53 reduced the size of tumor spheres in isogenic control H1975 cells (Figure 6E,F), indicating that mutant p53 contributes to tumor cell growth. Similarly, the size of tumor spheres was reduced in PolH-KO H1975 cells as compared to isogenic control cells (Figure 6E,F). Furthermore, the size of tumor spheres was further decreased by knockdown of mutant p53 in PolH-KO cells (Figure 6E,F), consistent with the observation that the level of mutant p53 protein was decreased most by combined PolH-KO and p53-knockdown (Figure 6D, compare lanes 2–3 with 5–6, respectively). Together, these findings suggest that PolH and p53 cooperate to promote tumor cell growth.

## 4. Discussion

PolH is a TLS polymerase and can mediate translesion DNA synthesis by enabling DNA replication across damaged DNA. As an error-prone DNA polymerase, PolH is found to assist tumor cells in escaping from DNA damage and therefore contribute to chemoresistance. In this study, we found that PolH modulates mutant p53 expression via SKP2 E3 ligase, which subsequently promotes proteasomal degradation of mutant p53 protein. We also showed that tumor cell growth and survival are suppressed by loss of PolH in part via decreased expression of mutant p53, which can be further suppressed by additional knockdown of mutant p53. Together, our data supported a model that PolH and mutant p53 cooperate to promote tumor progression and chemoresistance, and that the PolH–mutant p53 pathway can be explored as a therapeutic strategy for mutant p53-driven cancers. A model to elucidate these findings is proposed in Figure 6G.

Our study suggests SKP2 represses mutant p53 expression and subsequently promotes mutant p53 proteasome degradation (Figure 4 and Figure 5). Consistent with our data, a recent publication showed that knockdown of SKP2 leads to enhanced mutant p53 expression in A431 and A388 cells, both of which are human skin squamous cell carcinoma cell lines [45]. Notably, SKP2 has been previously identified as an oncogenic E3 ligase that promotes degradation of several important cell-cycle regulators, including the cyclin-dependent kinase inhibitors p21 and p27 [46,47,48]. Thus, it may appear paradoxical that SKP2 promotes degradation of mutant p53, which also functions as an oncogenic driver. However, these findings are not necessarily contradictory as the regulation of mutant p53 by SKP2 could be context-dependent. For example, the oncogenic E3 ligase MDM2, a key negative regulator of wild-type p53, has also been found to ubiquitinate and degrade mutant p53, albeit with lower efficiency than it degrades wild-type p53 [49,50]. Indeed, we also examined whether SKP2 regulated wild-type p53 expression in PolH-KO A549 cells. We found that although SKP2 was increased by PolH-KO in A549 cells, the level of wild-type p53 was not decreased (Supplemental Figure S1). These data suggest that SKP2 preferentially suppresses mutant p53. Moreover, in this study, we showed that SKP2 interacts with multiple p53 mutants, including R273H and R248W (Figure 5A–D). We also found that SKP2, but not F-box-deleted SKP2, promotes poly-ubiquitination of mutant p53(R273H) (Figure 5E,F). However, it remained to be determined whether SKP2 promotes proteasome degradation of other p53 mutants. Together, our findings provide a potential explanation for how PolH loss leads to reduced mutant p53 abundance and further studies are needed to elucidate whether SKP2 is an E3 ligase for both wild-type and mutant p53.

In our study, we found that loss of PolH leads to increased expression of SKP2 protein and transcript (Figure 3) although the underlying mechanism remains to be elucidated. These data suggest that PolH may exert additional functions. Indeed, a previous study showed that TLS polymerases participate in transcriptional regulation through interactions with transcription factor IIH (TFIIH) [51]. Thus, it is possible that PolH may recruit transcription factor IIH to upregulate SKP2 transcription. Moreover, loss of PolH is also known to induce DNA damage response, which can trigger activation of E2F1, which is known to transactivate SKP2 [52]. Thus, these possibilities need to be investigated in the future to elucidate how PolH modulates SKP2 transcription.

The results from this study suggest that PolH may contribute to tumor cell survival through a dual function: promoting tolerance to DNA damage while simultaneously maintaining mutant p53 protein stability and associated oncogenic signaling. The convergence of PolH and mutant p53 signaling could provide tumor cells with a selective advantage by enabling them to withstand persistent genotoxic stress while sustaining mutant p53-dependent proliferative and survival signaling, particularly in cancers harboring frequent TP53 mutations. Supporting this, we showed that although both PolH and mutant p53-deficiency inhibited tumor sphere formation, the inhibition was enhanced by combined knockdown of PolH and mutant p53 (Figure 6). These data indicate that while PolH and mutant p53 cooperate within a shared regulatory axis, their contributions to tumor growth are also non-redundant. However, it is important to note that mutant p53 is highly heterogeneous. The variants evaluated in this study (e.g., R273H and R248W) are primarily DNA-binding defective mutants. Therefore, it remains to be determined whether PolH deficiency similarly affects conformational mutant p53 variants, such as R175H. Nevertheless, because mutant p53 functions as a critical oncogenic driver in many cancers [34,35,36], our findings may have important biological consequences and therapeutic implications. Indeed, small-molecule inhibitors for PolH have been discovered and were found to sensitize cells to platinum-based chemotherapies [53,54]. Additionally, we and others have found that several small molecules, such as Arsenic Trioxide and HDAC inhibitors, repress mutant p53 expression [55,56,57]. Therefore, further studies are needed to test whether dual inhibition of the PolH and mutant p53 signaling pathways exerts a synergistic cancer-killing effect.

In conclusion, this study reveals a novel role for PolH in promoting tumor cell growth and survival by maintaining mutant p53 protein stability via SKP2. Our findings suggest that beyond its canonical function in translesion DNA synthesis and DNA damage tolerance, PolH sustains mutant p53-dependent oncogenic signaling, thereby providing an additional mechanism by which PolH contributes to tumor progression and chemoresistance. The PolH–SKP2–mutant p53 axis may therefore represent a potential therapeutic vulnerability, particularly in cancers harboring mutant p53. Further characterization of this pathway may provide new insights into the mechanisms linking DNA damage tolerance to oncogenic signaling and identify new therapeutic strategies for mutant p53-driven cancers.

## Figures and Tables

**Table 1:** p53 status in the cell lines used in this study.

Cell Line	TP53 Status	Notes
H1975	R273H	R273H and R248W are defective in DNA binding; P309S is not classified as structural/conformational mutant
CL1-5	R248W	
SW480	R273H/P309S	
MIA-PaCa-2	R248W	
A549	WT	

## Data Availability

All study data are included within the article.

## Supplementary Materials

The following supporting information can be downloaded at: https://www.mdpi.com/article/10.3390/cells15201881/s1. Figure S1: Loss of PolH has little effect on wild-type p53.
